# Statement of Retraction: The long non-coding RNA ASMTL-AS1 promotes hepatocellular carcinoma progression by sponging miR-1343-3p that suppresses LAMC1 (laminin subunit gamma 1)

**DOI:** 10.1080/21655979.2023.2269349

**Published:** 2023-10-18

We, the Editors and Publisher of the journal *Bioengineered*, have retracted the following article:

Yanjie Mou & Qinguo Sun (2022) The long non-coding RNA ASMTL-AS1 promotes hepatocellular carcinoma progression by sponging miR-1343-3p that suppresses LAMC1 (laminin subunit gamma 1), Bioengineered, 13:1, 746-758, DOI: 10.1080/21655979.2021.2012628

Since publication, significant concerns have been raised about the integrity of the data and reported results in the article. When approached for an explanation, the authors did not provide their original data or any necessary supporting information. As verifying the validity of published work is core to the integrity of the scholarly record, we are therefore retracting the article. All authors listed in this publication have been informed. The authors have not responded to notice of this retraction.

We have been informed in our decision-making by our ethical policies and the COPE guidelines.

The retracted article will remain online to maintain the scholarly record, but it will be digitally watermarked on each page as ‘Retracted’.

